# Rare and rarer: percutaneous transcatheter mitral commissurotomy using over-the-wire technique in a patient with mucopolysaccharidosis—a case report

**DOI:** 10.1093/ehjcr/ytag266

**Published:** 2026-07-06

**Authors:** Darshita Gosai, Sriram Easwaran, Ajaykumar Mahajan, Pratap Nathani, Milind Phadke

**Affiliations:** Department of Cardiology, LTMG Hospital, Mumbai 400022, India; Department of Cardiology, LTMG Hospital, Mumbai 400022, India; Department of Cardiology, Seth G S Medical College and KEM Hospital, Mumbai 400012, India; Department of Cardiology, LTMG Hospital, Mumbai 400022, India; Department of Cardiology, LTMG Hospital, Mumbai 400022, India

**Keywords:** Mucopolysaccharidosis, Over-the-wire technique, Mitral valvuloplasty, Valvular heart disease, Non-rheumatic mitral stenosis, Case report

## Abstract

**Background:**

Percutaneous transcatheter mitral commissurotomy (PTMC) by the Inoue technique is the traditional technique to relieve rheumatic mitral stenosis. Mucopolysaccharidoses, a group of rare lysosomal storage disorders, result in skeletal and vertebral anomalies and cardiac valvular abnormalities. The coexistence of both pathologies is rare, and mitral stenosis in such a scenario may pose significant technical challenges.

**Case summary:**

We present the case of a short statured 15-year-old girl with mucopolysaccharidosis Type VI with severe mitral stenosis and moderate aortic stenosis. Echocardiography revealed features suggestive of rheumatic involvement rather than due to the syndrome. Due to high surgical risk and unique anatomical characteristics, percutaneous approach was more amenable than surgery. The patient underwent successful PTMC using an unconventional over-the-wire technique instead of the traditional Inoue technique.

**Discussion:**

This case highlights the importance of a case-based approach in unique anatomic and high-risk situations for better outcomes, which may need out-of-the-box thinking.

Learning points

- Two rare conditions can coexist, raising questions about the relative contribution of each to the observed pathology. Imaging is mandatory for the detailed evaluation of valvular involvement, which can provide useful insights regarding aetiology and treatment.
- Treatment strategies always need individualization and team approach for better patient outcomes.

## Introduction

Mucopolysaccharidoses (MPSs) are a group of rare lysosomal storage disorders characterized by abnormal deposition of glycosaminoglycans (GAGs) in connective tissue, leading to skeletal and vertebral anomalies, corneal and retinal abnormalities, facial features, endocrine abnormalities, and cardiac valve and subvalvular pathology. Cardiac involvement is usually in the form of mitral valve prolapse (MVP) due to excessively thickened valves secondary to deposition of GAGs in the valve tissue and chordae tendineae, and it typically presents as valvular regurgitation. Occasionally, there may be cardiac conduction abnormalities. Though the bulky leaflets can impede valve outflow, commissural fusion is not a feature of these disorders, and symptomatic cases often require corrective surgery. Rheumatic heart disease characterized by commissural and chordal fusion in the mitral valve (MV) is still a commonly seen condition even today in developing and underdeveloped countries and usually presents with either valvular stenosis or regurgitation depending upon the stage of disease at which the diagnosis is made. However, the coexistence of both these aetiologies in a single patient has not been reported in literature till date.

## Summary figure

**Figure:**
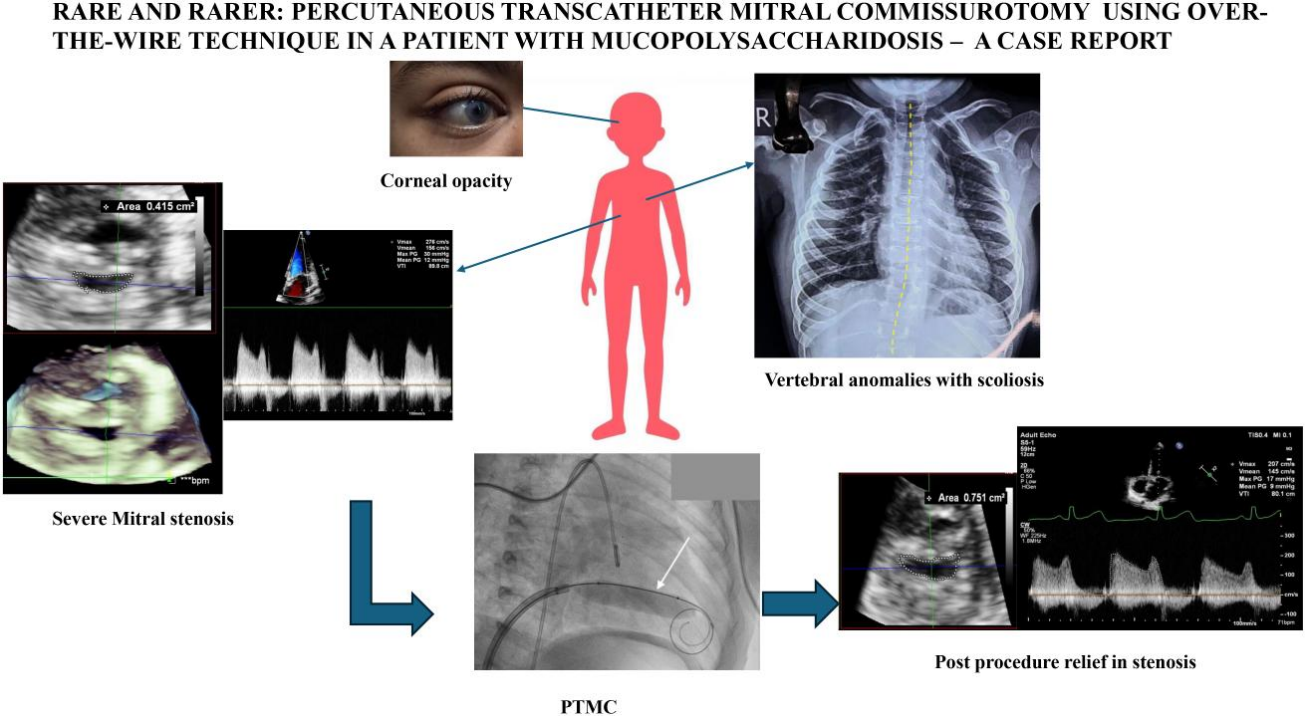


## Case presentation

**Figure 1:**
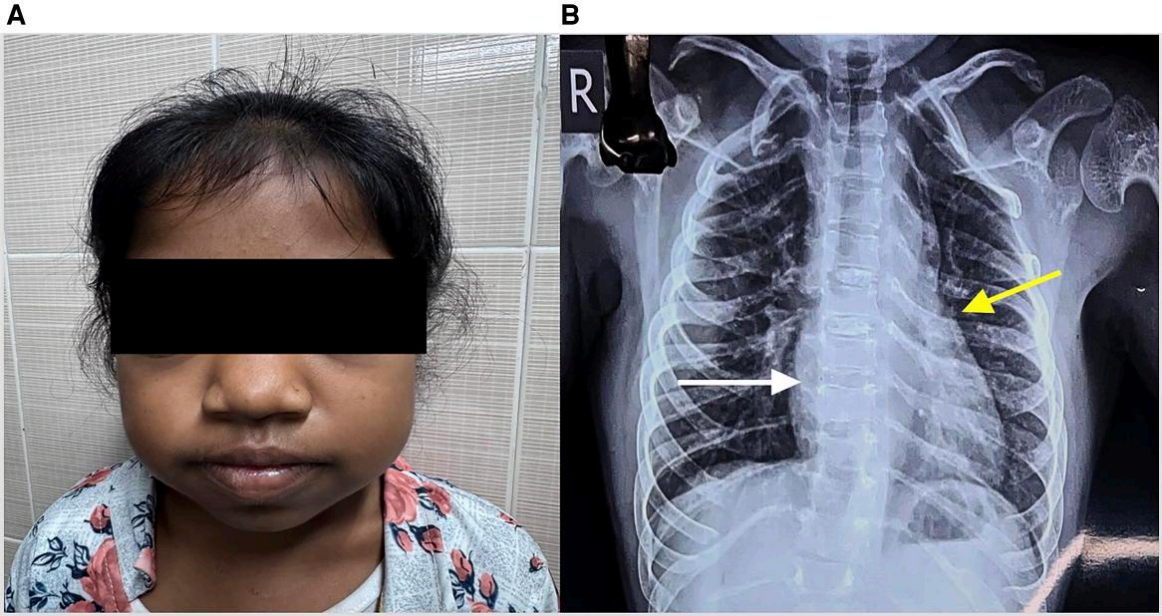
(*A*) Image of the child’s face showing characteristic facial features, such as flattened nasal bridge, short neck, thick lips, wide mouth, and sparse hair. (*B*) Chest X-ray posteroanterior (PA) view demonstrating the double atrial shadow (bottom white arrow) and straightened left heart border (top right arrow).

A 15-year-old girl presented with progressive dyspnoea, worsening from New York Heart Association (NYHA) Class II to NYHA Class III over 4 years. Physical examination was notable for short stature (both height and weight below the expected percentile) with coarse facial features, prominent forehead, deep set eyes, thick lips, broad mouth, and gingival hypertrophy (*Figure 1A*). She had peripheral corneal clouding, causing visual blurring. Other notable deformities noted were kyphoscoliosis, pectus excavatum, and radial, humeral, and ulnar deformities restricting the range of motion. Cardiac auscultation was notable for normal heart sounds and a Grade III/VI systolic murmur at the second right intercostal space and long mid-diastolic murmur at the apex.

The electrocardiogram showed sinus rhythm, normal QRS axis, and left atrial enlargement. The chest radiogram showed a typical ‘double density’ left atrial shadow, straightening of the left heart border, and dilated main pulmonary artery (*Figure 1B*).

Important diagnostic considerations included rheumatic heart disease, congenital MV disease, and syndrome-associated valvular disease (e.g. mucopolysaccharidosis)

**Figure 2:**
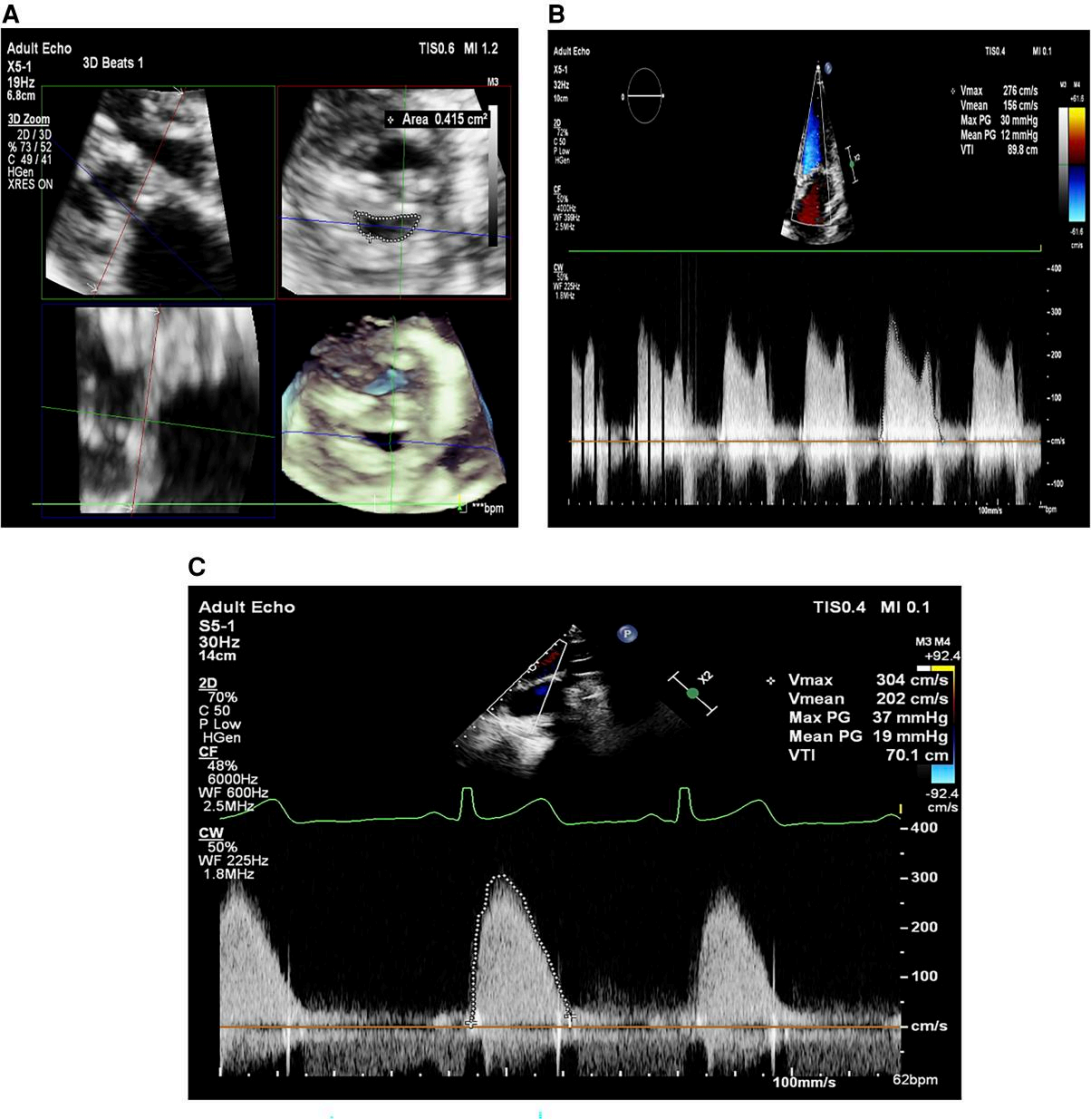
(*A*) Baseline multiplanar three-dimensional reconstruction of the mitral valve orifice on transthoracic echocardiography demonstrating an effective valve area of 0.41 cm^2^, suggesting critical mitral stenosis (upper right segment). (*B*) Continuous wave Doppler study across the mitral valve demonstrating a severe mean transvalvular gradient of 12 mmHg. (*C*) Continuous wave Doppler across the aortic valve in the right parasternal view demonstrating a mean transvalvular gradient of 19 mmHg and a peak velocity of 3.04 m/s suggestive of mild aortic stenosis.

The transthoracic echo revealed moderate thickening of the mitral and aortic valves, leading to reduced leaflet mobility, with mitral commissural fusion, notably free of significant calcium and moderate subvalvular thickening. The MV Doppler study demonstrated a peak gradient of 30 mmHg and a mean gradient of 12 mmHg (*Figure 2B*) with an area of 0.415 cm^2^ by three-dimensional (3D) planimetry (*Figure 2A*). The aortic valve Doppler study showed a peak velocity of 3.1 m/s, a peak gradient of 40 mmHg, and a mean gradient of 22 mmHg (*Figure 2C*), the and aortic valve area (AVA) by continuity equation was 1.6 cm^2^. The Wilkin score was 8/16. She had mild mitral regurgitation (MR), mild tricuspid regurgitation (TR), and peak pulmonary artery systolic pressure (PASP) by a TR jet of 42 mmHg.

Routine investigations including complete haemogram, liver, and renal biochemistries and thyroid profile were normal. Her skeletal X-ray survey revealed codfish-shaped vertebrae, femoral head erosion, deformities in bilateral radii and ulnae, and bilateral hooked humeri. The skull showed an enlarged sella turcica and anterior clinoid process erosion. Also notable were slender ribs and irregular acetabular margins.

Whole genome sequencing revealed homozygous missense variant in exon 8 of ARSB gene typically seen in MPS Type VI (Maroteaux–Lamy syndrome)

In view of the uniqueness due to short stature, low body surface area (0.79 m^2^), and a complex valvular anatomy, with NYHA Class III symptoms despite optimal diuretic dosage, our heart team took a decision to plan a percutaneous relief of the mitral stenosis (MS) with a submaximal balloon inflation due to a high surgical risk. Percutaneous transcatheter mitral commissurotomy (PTMC) using an over-the-wire (OTW) valvuloplasty technique commonly used in balloon dilatation of aortic and pulmonary valves was considered a better alternative to the more commonly used Inoue technique was planned.^1^

**Figure 3:**
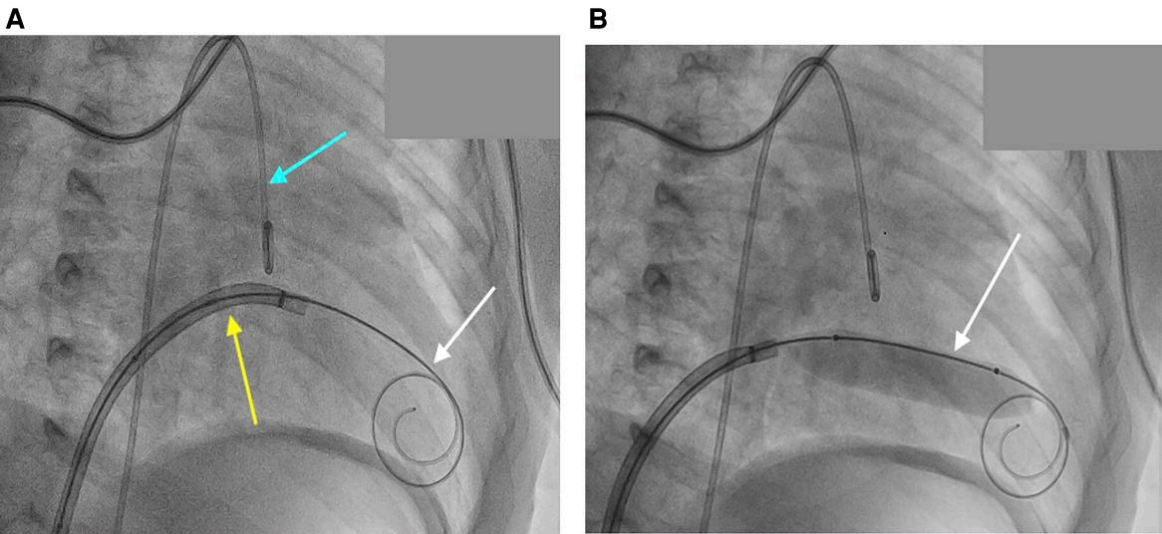
(*A*) Fluoroscopy in the right anterior oblique (RAO) view demonstrating pigtail in non-coronary cusp (NCC) (blue arrow), Agilis sheath (yellow arrow), and Safari extra small wire (white arrow). (*B*) The right anterior oblique view with the Atlas Gold balloon across the mitral valve over the wire (white arrow).

**Figure 4:**
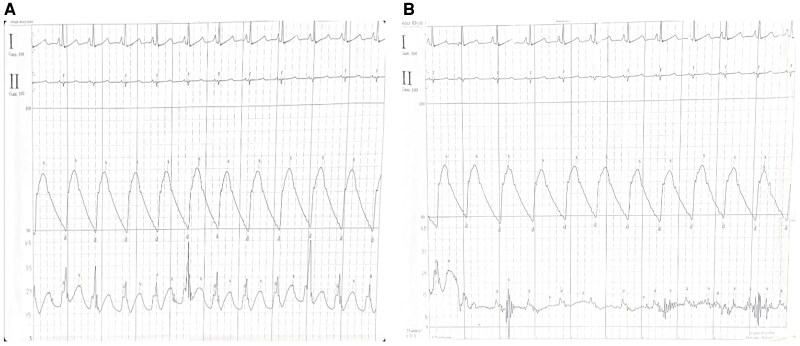
(*A*) Pre-procedure pressure tracing showing a mean left atrial pressure of 15 mmHg. (*B*) Pressure tracing of the left atrium demonstrating a mean gradient of 7 mmHg post-mitral valvuloplasty.

**Figure 5:**
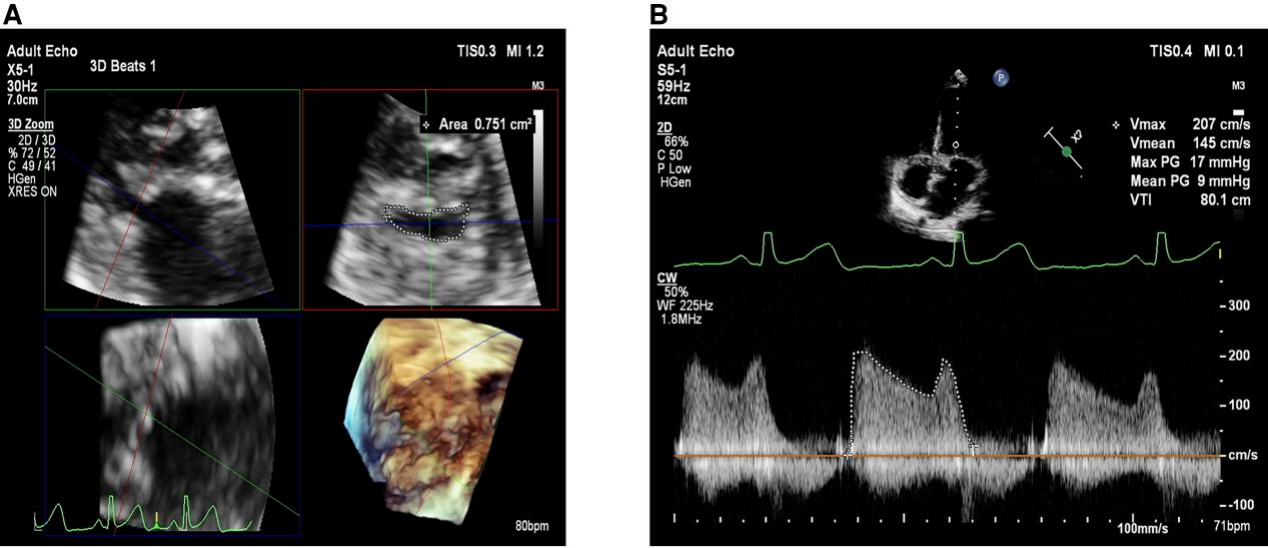
(*A*) Baseline multiplanar three-dimensional reconstruction of the mitral valve orifice on transthoracic echocardiography post-procedure demonstrating an effective valve area of 0.75 cm^2^, suggesting doubling of the previous valve area in *Figure 2A* (upper right segment). (*B*) Continuous wave Doppler study across the mitral valve demonstrating a severe mean transvalvular gradient of 9 mmHg.

Under general anaesthesia, a standard transseptal puncture was taken under fluoroscopic guidance at the fossa ovalis. An 0.035 spring guide wire (Suretech Medical Ltd.) was placed in the left atrium (LA). An 8-F steerable Agilis sheath (Abbott Vascular) was advanced over the spring guide wire into the LA. Through this sheath, a 6-F pigtail catheter was advanced over an 0.035 double-length J-tipped wire. The Agilis sheath was flexed using the flexor knob to make it coaxial with the MV orifice, and the valve was crossed with the pigtail catheter to avoid chordal entrapment. An 0.035 double-length J-tipped wire was exchanged for a Safari extra small (XS) wire (Boston Scientific), which was positioned in the left ventricular apex (*Figure 3A*). A 12 mm * 40 mm Atlas Gold balloon (Bard Peripheral Vascular) was advanced over the wire across the diseased MV. A single submaximal inflation was given with relief of the balloon waist (*Figure 3B*), and a significant drop was noted in the mean LA pressure from 20 to 9 mmHg (*Figure 4A* and *B*). On echocardiography, there was an increase in MV area to 0.751 cm^2^ by 3D planimetry and Doppler analysis demonstrated a more than 50% fall in the mean gradient to 9 mmHg (*Figure 5A* and *B*), with no increase in the pre-existing mild MR.

Although the patient experienced significant symptomatic relief following the procedure, keeping in mind that this was not the definitive treatment, the parents were counselled regarding the need for regular follow-up. They were made aware of the eventual need for surgical therapy for the valves. She was discharged on an optimal diuretic dosage and advised to follow up in 6 months, even though a telephonic follow-up at 4 months confirmed her continued well-being.

## Discussion

Mucopolysaccharidoses are a group of lysosomal storage disorders caused by deficiencies in enzymes required for breakdown and turnover of GAGs. As a result, these large sugar molecules essential for connective tissue stability accumulate in blood, fluids, and soft tissue sites throughout the body.

Typical features include dwarfism (i.e. growth retardation), skeletal and joint deformities, dysmorphic facial features, central nervous system involvement (including mental retardation), ocular (corneal clouding, retinal degeneration, and blindness) and hearing impairment, respiratory difficulties, gastrointestinal pathology, and umbilical or inguinal hernia.

Amongst cardiovascular manifestations, most studies have reported valvular regurgitation to be more common than stenosis, with predominantly MV involvement.^2,3^

In our case, both mitral and aortic valves were simultaneously involved, but instead of the expected involvement by MPS, both aortic and MV leaflets demonstrated commissural fusion, MV chordal shortening and thickening, and reduced posterior mitral leaflet mobility with preserved mobility of anterior mitral leaflet, which are characteristic of rheumatic origin. Moreover, in underdeveloped and developing countries, rheumatic heart disease (RHD) is the most common cause of combined MS and aortic stenosis in young individuals.

Cardiac valve surgery is amongst the procedures frequently performed in patients with MPS, since they usually present with regurgitant lesions.^2,4^

In view of MS being the dominant lesion, PTMC was the procedure of choice considering the patient’s age, body surface area, anatomy, and rheumatic involvement of the MV. The Inoue technique is regarded as the gold standard for PTMC due to balloon characteristics, such as self-positioning configuration, size adjustability, and rapid inflation–deflation.^1^ However, the smallest available Inoue/Accura balloon is of 20 mm diameter, which would have been too large for our short statured patient with a small body surface area. Other challenges included very critical MS with a small valve orifice and small LA. Hence, we chose the rather unconventional OTW technique, using a 12 mm balloon, which was more suitable for the patient’s anatomy. Moreover, crossing the valve with a pigtail catheter can help in avoiding chordal engagement and help position the wire at the left ventricle (LV) apex. We aimed for submaximal balloon inflation due to a crowded subvalvular apparatus, with the sole aim of providing symptomatic relief.

The OTW technique, first described by Meier, *et al*. in 1992,^5^ included parking a stiff wire in the LV, followed by tracking of the PTMC balloon over the stiff wire.^5^ The OTW technique is useful in situations with difficult LV entry, such as low septal puncture, thick interatrial septum, LA/LA appendage clot, critical MS, giant LA perforation, injury to the mitral chordal apparatus, and the risk of ventricular arrhythmia. Although ventricular premature beats and non-sustained ventricular tachycardia can occur, they are usually transient, and haemodynamic compromise is not common. The presence of mechanical aortic prosthetic valve, LV mass, or thrombus is a contraindication for this technique.^6^

Despite all that can be done as a temporary measure for the patient, we still need to keep in mind that the definitive diagnosis will only be confirmed by a tissue diagnosis done during surgery. Such unique cases demand close follow-up, out-of-the-box thinking, and individualized therapy. We also need to be aware of the eventuality of the need for a surgical valve replacement in the future and genetic and therapeutic counselling for the parents. With ongoing research in the field of MPS, early diagnosis and initiation of enzyme therapies are also avenues to be explored in the future.

Individualized interventional strategies should be used according to patient-specific anatomical and clinical considerations, in rare clinical and anatomic scenarios.

## Conclusions

This case describes unique coexistence of cardiac involvement of mucopolysaccharidosis Type IV with rheumatic heart disease. The case also enforces the concept of patient-specific individualization of treatment strategies to achieve better outcomes.

## Supplementary Material

ytag266_Supplementary_Data

## Data Availability

The data underlying this article are available in the article and in its online supplementary material.

## Lead author biography

Dr Darshita Gosai is a third-year cardiology resident in the Department of Cardiology at Lokmanya Tilak Medical College, Mumbai. Her areas of special interest include interventional cardiology and echocardiography.
